# The relationship of homocysteine and its redox forms with renal toxicity after high-dose methotrexate treatment in patients with leukemia or lymphoma

**DOI:** 10.3389/fphar.2026.1972047

**Published:** 2026-10-20

**Authors:** Shuai-Shuai Gao, Chun-Ling Wu, Zheng-Yang Liu, Jin-Xuan Zheng, Hong-Yu Zhou, Jin-Yan Xie, Jia-Liang Guo, Wei-Chong Dong

**Affiliations:** 1 Department of Pharmacy, The Second Hospital of Hebei Medical University, Shijiazhuang, Hebei, China; 2 School of Pharmacy, Hebei Medical University, Shijiazhuang, Hebei, China; 3 Department of Orthopedics, Hebei Medical University Third Hospital, Shijiazhuang, China

**Keywords:** hepatotoxicity, homocysteine, homocystine, methotrexate, nephrotoxicity, reduced homocysteine

## Abstract

**Background:**

High-dose methotrexate (HD-MTX) chemotherapy inhibits dihydrofolate reductase, leading to impaired homocysteine metabolism, elevated levels, and associated clinical adverse reactions. Currently, studies often monitor total homocysteine (tHcy) levels, but its toxic mechanism remains unclear.

**Objectives:**

Given the association between HD-MTX toxicity and oxidative stress, this study aimed to analyze the correlations between tHcy and its redox forms with liver/kidney function indicators, and to evaluate their discriminatory ability for hepatic and renal toxicity.

**Methods:**

A total of 310 blood samples were collected from 89 patients (38 adults, 51 children) with leukemia or lymphoma treated with HD-MTX. Concentrations of tHcy, free reduced homocysteine (Hcy), free oxidized homocysteine (HHcy, homocystine), and the ratio of Hcy concentration to HHcy concentration (Hcy/HHcy) were measured at 42, 72, and 96 h post-chemotherapy. Generalized estimating equations (GEE) were used to assess associations between tHcy-related redox forms and liver/kidney function indicators, and to compare concentration differences between toxicity and non-toxicity groups. Receiver operating characteristic (ROC) curve analysis was applied to evaluate the discriminatory ability of relevant indicators for HD-MTX-related toxicity.

**Results:**

In pediatric patients, Hcy/HHcy was significantly negatively correlated with creatinine (CR) (*P* < 0.028), and tHcy was significantly negatively correlated with alanine aminotransferase (ALT), total bilirubin (TB), and direct bilirubin (DB). No significant associations were observed in adult patients. Compared with the control group, the kidney injury group showed significant differences in Hcy and HHcy levels at 42 h (*P* < 0.05), Hcy at 72 h, and HHcy at 96 h. No significant differences were observed in the liver injury group. HHcy, tHcy, and Hcy/HHcy at 42 h all demonstrated good discriminatory ability for kidney injury, with HHcy showing an AUC of 0.817 (95% CI: 0.702–0.932, *P* < 0.01). tHcy at 72 h and 96 h showed reverse discriminatory ability for liver and kidney injury.

**Conclusion:**

Alterations in tHcy redox status after HD-MTX treatment, particularly changes in HHcy levels and the Hcy/HHcy, may be associated with renal function changes and may have potential value for identifying patients with HD-MTX-related nephrotoxicity.

## 1 Introduction

High-dose methotrexate (HD-MTX) is a standard treatment regimen for various malignancies, including acute lymphoblastic leukemia, malignant lymphoma, and osteosarcoma (Ogawa et al., 2024). However, this therapy is frequently associated with a range of toxic effects, including nephrotoxicity, mucositis, hepatotoxicity, and neurotoxicity (Choi et al., 2024). Among these, long-term or high-dose MTX use may lead to hepatitis or even cirrhosis (Roenigk et al., 1988). MTX is primarily excreted unchanged via the kidneys, with a small portion metabolized in the liver to 7-hydroxymethotrexate (7-OH-MTX) (Fabresse et al., 2018). 7-OH-MTX is also cleared renally, but at a slower rate. Furthermore, in acidic environments, the solubility of 7-OH-MTX is only one-third to one-fifth that of MTX, making it more prone to crystallization and precipitation within renal tubules, thereby causing renal damage (Bing et al., 2025). Renal toxicity impairs renal clearance function; prolonged exposure to toxic concentrations further deteriorates renal function, exacerbates non-renal adverse events, and can lead to patient mortality in severe cases (Howard et al., 2016). MTX exhibits considerable variability in pharmacokinetics and pharmacodynamics, characterized by a narrow therapeutic window that can lead to serious adverse reactions. As a result, therapeutic drug monitoring is essential to ensure medication safety. Previous studies have found a link between blood levels of methotrexate and its metabolite 7-OH-MTX with hepatic and renal toxicity, (Csordas et al., 2013; Mei et al., 2020; Yang et al., 2018), but the exact mechanisms remain uncertain.

Methotrexate reduces intracellular levels of reduced folate by targeting dihydrofolate reductase inhibition (Goldman and Matherly, 1985). 5-Methyltetrahydrofolate, a form of reduced folate, plays a crucial role in the conversion of total homocysteine (tHcy) to methionine (Figure 1) (Kubota et al., 2014).

**FIGURE 1:**
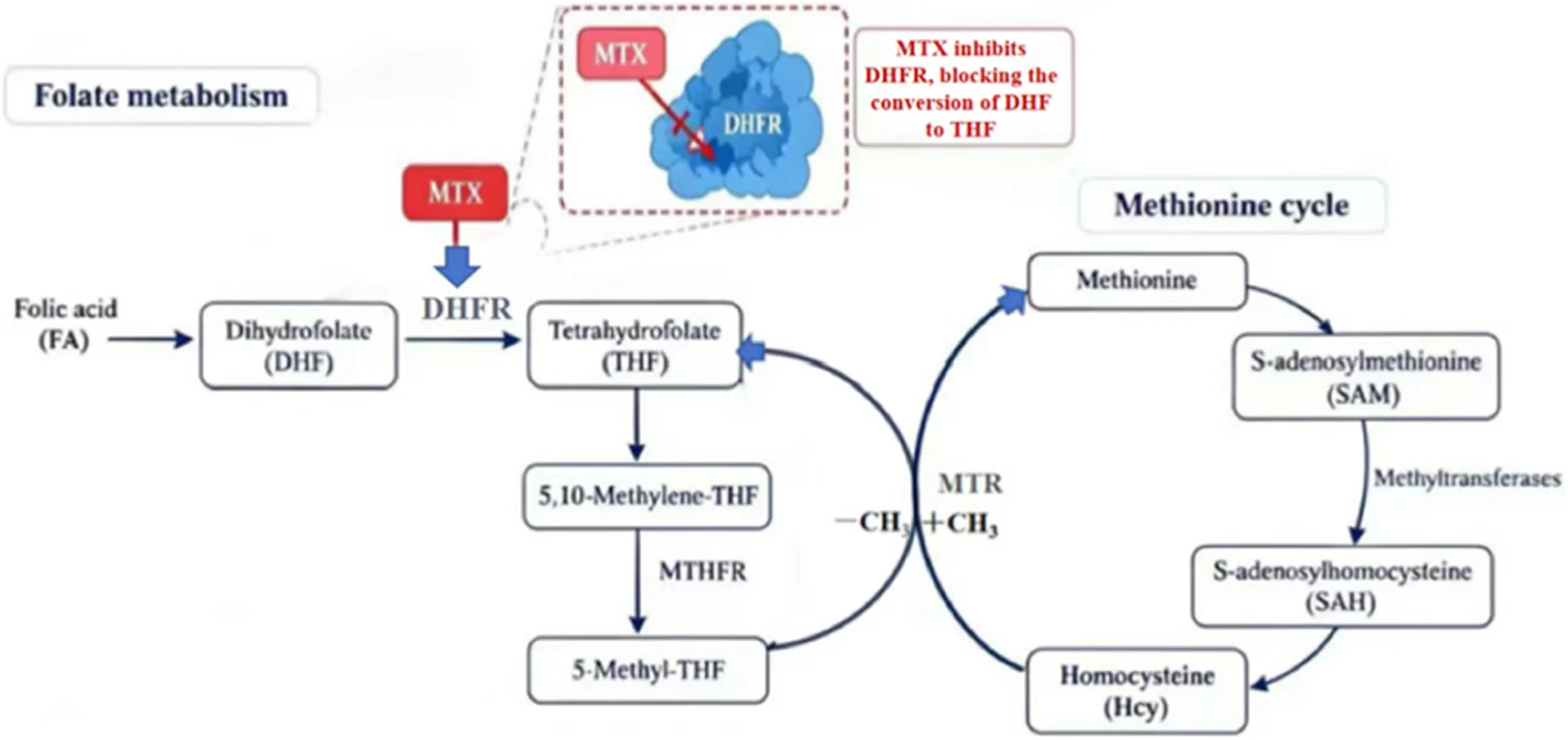
The metabolic pathway of homocysteine in the context of MTX-induced folate antagonism. DHFR is dihydrofolate reductase.

Therefore, the use of MTX, especially HD-MTX, disrupts homocysteine metabolism and results in increased concentrations of this amino acid. Aminothiols are biologically active compounds that play vital roles in redox regulation, DNA synthesis, apoptosis inhibition, metastasis, and angiogenesis (Dong et al., 2021). Homocysteine, a small-molecule amino thiol, induces apoptosis in vascular endothelial cells by increasing reactive oxygen species (ROS) production and generating an abundance of oxidative free radicals, thereby causing endothelial damage (Ostrakhovitch and Tabibzadeh, 2019). Its serum levels are directly or indirectly associated with various diseases, including cardiovascular disorders, neurological conditions, diabetes, and malignant tumors (Ren et al., 2020). Studies indicate that HD-MTX chemotherapy causes transient elevations in tHcy levels, potentially associated with seizures and hepatotoxicity in children with acute lymphoblastic leukemia (Kubota et al., 2014; Kishi et al., 2003). Furthermore, MTX toxicity scores showed significant correlation with elevated plasma tHcy concentrations within 48 h post-administration (*P* = 0.02), while high tHcy levels were also associated with increased MTX plasma concentrations within 24 h. (Kubota et al., 2014; Mahmoud et al., 2018). Other literature indicates that plasma tHcy levels correlate with the occurrence of HD-MTX neurotoxicity (Kubota et al., 2014). In rheumatoid arthritis patients, MTX-induced hepatotoxicity is associated with elevated plasma tHcy, and this elevation can be prevented by folate supplementation (Hornung et al., 2004). However, plasma tHcy levels after HD-MTX treatment are not associated with the occurrence of mucositis (den Hoed et al., 2015).

All current studies have measured tHcy concentration, and the specific mechanisms by which it mediates toxicity remain unclear. Plasma tHcy is a mixture of free reduced homocysteine (Hcy), free oxidized homocysteine (HHcy), and various bound forms (Kishi et al., 2003). Oxidative stress represents a state of disturbed equilibrium between ROS production and antioxidant efficiency, and is associated with numerous diseases (Pisoschi and Pop, 2015). A dynamic equilibrium exists between the free concentrations of reduced and oxidized tHcy in plasma, and its disruption is considered a key indicator of chronic oxidative stress (Escoba et al., 2016). Studies indicate that HD-MTX induces significant oxidative stress and increases apoptosis (Taylor et al., 2015), while MTX-related hepatotoxicity and renal injury are also closely associated with oxidative damage and inflammatory responses (Deniz et al., 2025; Hassanein et al., 2023; Hassanein et al., 2019; Morsy et al., 2022). Therefore, we further focused on the relative levels between Hcy and its redox form HHcy, and explored the potential association of the ratio of Hcy concentration to HHcy (Hcy/HHcy) with HD-MTX treatment-related hepatorenal toxicity. To date, no studies have utilized the Hcy/HHcy to investigate HD-MTX chemotherapy toxicity.

This study aimed to explore the potential relationships between tHcy, Hcy, HHcy, and the Hcy/HHcy and HD-MTX chemotherapy-related hepatic and renal abnormalities. Specific objectives include: (1) Correlating concentrations with hepatic and renal indicators; (2) Comparing concentration differences between liver injury and normal groups, as well as kidney injury and normal groups; (3) Evaluating the discriminatory performance of these measures for hepatotoxicity and nephrotoxicity using receiver operating characteristic (ROC) curve analyses.

## 2 Experimental

### 2.1 Sample collection

This study complies with the requirements of the Declaration of Helsinki, and the study protocol has been approved by the Medical Ethics Committee of the Second Hospital of Hebei Medical University (Approval No.: 2024-R095). All participants (or their legal guardians, in the case of minors) have signed written informed consent forms, indicating their voluntary participation in this study. A total of 89 patients with lymphoma and acute lymphoblastic leukemia (ALL) (38 adults and 51 children) who received HD-MTX at the Second Hospital of Hebei Medical University from June 2024 to August 2025 were included, and 310 blood samples were collected. According to *the Chinese guidelines for the diagnosis and treatment of acute lymphoblastic leukemia or lymphoma in children (2018 edition)*, all pediatric patients received HD-MTX at a dose of 2–5 g/m^2^. For adult patients, according to the *Chinese Guidelines for the Diagnosis and Treatment of Adult Acute Lymphoblastic Leukemia (2024 edition)*, the recommended dose range of HD-MTX is 1–5 g/m^2^, with a dose of 5 g/m^2^ applicable to patients with T-ALL. The plasma MTX concentration was monitored at 42 h post-infusion, and the LV rescue dose was adjusted according to the MTX concentration. Rescue therapy was administered every 6 h until the MTX concentration fell below 0.25 μmol·L^-1^ at 42 h for pediatric patients, or below 0.1 μmol·L^-1^ at 72 h for adult patients. Hydration: starting 12 h before administration, with a daily hydration volume of ≥2500–3000 mL/m^2^, continued for 72 h or longer until MTX<0.1 μmol·L^-1^. Urine alkalinization: urine pH > 7.0 before chemotherapy, and urine pH maintained between 7.0 and 8.0 during chemotherapy. A total of 310 blood samples from 89 patients, including repeat tests from the same patient within a single treatment cycle as well as repeat tests across different treatment cycles.

Blood samples were collected at 42, 72, and 96 h after HD-MTX administration. The tHcy-related redox forms were measured synchronously at 42, 72, and 96 h after HD-MTX administration. All patients had normal hepatic and renal function indicators before HD-MTX. Hepatotoxicity and nephrotoxicity following HD-MTX administration were assessed according to the National Cancer Institute Common Terminology Criteria for Adverse Events (NCI-CTCAE), version 5.0. Hepatotoxicity was primarily assessed based on alanine aminotransferase (ALT) elevation, with aspartate aminotransferase (AST) and total bilirubin (TB) levels considered as additional indicators. Nephrotoxicity was assessed based on creatinine (CR) elevation.

According to the CTCAE v5.0 criteria, in our study, nephrotoxicity was defined as CR level exceeding the laboratory upper limit of normal (>ULN). Hepatotoxicity was defined as an ALT level exceeding the ULN, together with an AST or TB level exceeding the ULN. Laboratory abnormalities meeting the above criteria and corresponding to CTCAE Grade ≥1 were considered hepatotoxicity or nephrotoxicity events and were included in subsequent statistical analyses. The ULN for the laboratory parameters was determined according to the reference ranges provided by the Clinical Laboratory of the Second Hospital of Hebei Medical University.

### 2.2 Determination of tHcy, Hcy, and HHcy concentration

Peripheral venous blood samples were collected in EDTA-containing anticoagulant tubes at 42, 72, and 96 h after HD-MTX. Immediately transfer 900 μL of whole blood to the HFCF-UF device, which has been pre-filled with 100 μL of a 100 mmol·L^-1^ NEM solution and 10 μL of an internal standard working solution (3.6 mmol·L^-1^ Hcy-D_4_-NEM), to prevent the auto-oxidation of thiols. Allow the mixture to react at room temperature for 1 min. Subsequently, centrifuge at 2.40 × 10^3^ g for 5 min at 4 °C, and collect the ultrafiltrate from the hollow fiber chamber using a syringe. Mix 30 μL of the ultrafiltrate with 30 μL of 2% sulfosalicylic acid, and inject 10 μL of the mixture into an HPLC-MS/MS system for analysis. Centrifuge the remaining whole blood at 2.40 × 10^3^ g for 5 min to obtain plasma, and determine the concentration of tHcy in the plasma using an enzymatic cycle method (Dong et al., 2021; Dong et al., 2023). The resulting samples can be analyzed immediately or stored in batches at −80 °C, avoiding repeated freeze-thaw cycles; thaw at 4 °C prior to analysis.

The analytical methods used for Hcy and HHcy quantification were systematically validated. The validation parameters included calibration range, linearity, lower limit of quantification (LLOQ), precision, accuracy, recovery, matrix effects, and analyte stability. The detailed validation results were as follows: Hcy-NEM showed good linearity over the concentration range of 0.05–5 μmol·L^−1^, with a regression equation of Y = 4.06 × 10^4^X − 1.19 × 10^3^ (*r* ^2^ = 0.9976). HHcy showed good linearity over the concentration range of 0.004–0.04 μmol·L^−1^, with a regression equation of Y = 3.74 × 10^5^X + 2.08 × 10^3^ (*r* ^2^ = 0.9969). The LLOQs for Hcy-NEM and HHcy were 0.05 μmol·L^−1^ and 0.004 μmol·L^−1^, respectively. Accuracy and precision were evaluated using quality control samples at multiple concentration levels. The relative recoveries ranged from 92.36% to 111.48% for Hcy-NEM and from 92.35% to 105.93% for HHcy. Both intra-day and inter-day precision demonstrated good reproducibility, with RSD values below 10%. Extraction recovery and matrix effects were also evaluated. The extraction recoveries for Hcy-NEM ranged from 95.65% to 103.43%, while those for HHcy ranged from 88.78% to 108.10%. The matrix effect assessment showed that the CV values for both analytes were below 10%, indicating no significant matrix interference. In addition, we evaluated room-temperature stability, freezing stability, freeze–thaw stability, and post-processing stability. QC samples were maintained at room temperature (26 °C) for 12 h, subjected to three freeze–thaw cycles, stored at −40 °C for 14 days, or kept in the autosampler at 15 °C for up to 24 h after processing. All QC samples remained stable under these conditions, with RSD values below 13%.

### 2.3 Statistical analysis

Statistical analyses were performed using SPSS version 27.0 (IBM Corp., Armonk, NY, USA). Multivariable generalized estimating equation (GEE) models were used to evaluate the associations between tHcy-related parameters (tHcy, Hcy, HHcy, and Hcy/HHcy) and liver function indicators (ALT, AST, TB, DB, IB) and kidney function indicators (CR, CCR), with pediatric (<16 years) and adult (≥16 years) patients analyzed separately. All models were adjusted for sex, age, height, body weight, baseline renal function indicators, baseline hepatic function indicators, MTX concentration, HD-MTX chemotherapy dose and delayed MTX elimination,and the resulting *P* values were corrected for multiple comparisons using the false discovery rate (FDR) method. GEE models were also used to compare tHcy-related parameter levels between the injury and non-injury groups for both hepatic and renal toxicity, with the same adjustments and FDR correction. As an exploratory analysis, ROC curves were generated to assess the discriminative ability of tHcy-related parameters for HD-MTX-associated hepatic and renal toxicity, and the area under the curve (AUC) with 95% confidence intervals (CIs) and optimal threshold values were calculated.

## 3 Results

### 3.1 Sample characteristics

This study included a total of 89 patients (38 adults and 51 children), with 310 blood samples collected and analyzed, including repeat tests from the same patient within a single treatment cycle as well as repeat tests across different treatment cycles. The repeated-measurement structure is shown in Supplementary Figure S1. The distribution of samples by collection time point was as follows: 143 samples at 42 h (Renal toxicity:12, Hepatic toxicity:52), 110 samples at 72 h (Renal toxicity:17, Hepatic toxicity:23), and 57 samples at ≥ 96 h (Renal toxicity:11, Hepatic toxicity:12). The clinical characteristics of the patients are shown in Table 1. For the estimation of CCR in children, the Schwartz formula was used: CCR (mL/min) = 36.5 × (height in cm/serum creatinine in μmol·L^-1^). For adults, CCR was calculated using the following formula: CCR (mL/min) = [(140 - age) × weight (kg)]/[0.818×serum creatinine (μmol·L^-1^)]. For women, this calculation adjusted the factor to 0.85. Baseline and clinical characteristics of patients with and without renal or hepatic toxicity are presented in Supplementary Tables S1, S2, respectively.

**TABLE 1:** The characteristics and corresponding concentrations of 310 clinical plasma samples with all sampling times.

Characteristics		Median (*P*_25_, *P*_75_)
Age (years)		13 (9.00, 37.00)
Body weight (kg)		45 (24.75, 69.00)
Body height (cm)		159 (130.00, 168.00)
Body surface area (BSA)		1.43 (0.93, 1.77)
Gender (male/female)		198/112
Creatinine, CR (μmol·L^-1^)		41.00 (29.98, 60.00)
Creatinine clearance, CCR		137.56 (102.77, 161.27)
Alanine transaminase, ALT (U/L)		42.70 (22.33, 74.18)
Aspartate aminotransferase, AST (U/L)		33.00 (22.90, 57.90)
Total bilirubin (TB)		11.50 (8.40, 17.33)
Direct bilirubin (DB)		4.00 (2.72, 6.10)
Indirect bilirubin (IB)		7.40 (5.40, 10.70)
Delayed elimination, n (%)		112 (36.12%)
Baseline ALT concentration (U/L)		35.77 (28.50, 37.25)
Baseline AST concentration (U/L)		30.1 (28.00, 33.10)
Baseline CR (μmol·L^-1^)		43.50 (41.42, 45.50)
Baseline CCR		133.23 (117.10, 160.88)
Baseline DB concentration		4.55 (3.51, 6.10)
Baseline TB concentration		9.51 (8.52, 13.90)
Baseline IB concentration		8.37 (6.52, 9.56)
MTX concentration (μmol·L^-1^)		0.12 (0.05, 0.28)
Patient disease classification (ALL/lymphoma)		66/23
Dose (g/m^2^)		3.54 (2.14, 4.58)
42 h, *n* = 143	tHcy concentration (μmol·L^-1^)	8.53 (5.80, 12.11)
​	Hcy concentration (μmol·L^-1^)	0.601 (0.28, 1.28)
​	HHcy concentration (μmol·L^-1^)	0.017 (0.006, 0.035)
​	Hcy/HHcy concentration	32.85 (15.23, 116.26)
72 h, *n* = 110	tHcy concentration (μmol·L^-1^)	10.13 (7.39, 15.00)
​	Hcy concentration (μmol·L^-1^)	0.672 (0.28, 1.67)
​	HHcy concentration (μmol·L^-1^)	0.017 (0.006, 0.035)
​	Hcy/HHcy concentration	36.38 (11.90, 119.14)
≥96 h, *n* = 57	tHcy concentration (μmol·L^-1^)	9.61 (6.81,14.83)
​	Hcy concentration (μmol·L^-1^)	0.682 (0.24, 1.38)
​	HHcy concentration (μmol·L^-1^)	0.020 (0.012, 0.043)
​	Hcy/HHcy concentration	19.77 (8.79, 110.24)

*P*_25_: 25th percentile, *P*_75_: 75th percentile.

### 3.2 The correlation analysis results

The results of the GEE analyses are presented in Table 2. In pediatric patients, Hcy/HHcy showed a significant negative association with the renal function indicator CR (*P* = 0.004, FDR-*P* = 0.028, 95% CI: −0.012∼-0.002). Additionally, tHcy was significantly negatively associated with ALT (95% CI: -6.151∼-0.624), TB (95% CI: −0.387∼-0.070), and DB (95% CI: −0.270∼-0.041) (FDR-*P*<0.05). However, no significant associations were observed among any tHcy-related parameters and hepatic or renal function indicators in adult patients (Table 3).

**TABLE 2:** GEE analysis of the associations between homocysteine and its redox forms and hepatic and renal function indicators in 51 pediatric patients.

Item		β	95% CI	*P* value	FDR-adjusted *P*
HCY	AST	3.366	−4.523∼11.255	0.403	0.779
	ALT	7.705	−4.937∼20.347	0.232	0.779
	DB	0.550	−2.580∼0.368	0.729	0.779
	TB	0.136	−0.536∼0.808	0.692	0.779
	IB	0.068	−0.407∼0.543	0.779	0.779
	CR	−1.131	−2.669∼0.408	0.150	0.779
	CCR	1.654	−2.687∼5.995	0.455	0.779
HHCY	AST	44.749	−166.158∼255.656	0.678	0.678
	ALT	82.258	−260.232∼424.749	0.638	0.678
	DB	−3.641	−8.964∼1.681	0.180	0.252
	TB	−16.752	−35.552∼2.049	0.081	0.233
	IB	−12.232	−29.549∼5.086	0.166	0.252
	CR	93.521	−14.738∼201.780	0.090	0.233
	CCR	−186.615	−409.209∼35.979	0.100	0.233
HCY/HHCY	AST	−0.019	−0.038∼-0.001	0.041	0.096
	ALT	−0.014	−0.050∼0.022	0.453	0.529
	DB	0.00024	−0.001∼0.001	0.606	0.606
	TB	0.001	−0.001∼0.003	0.317	0.444
	IB	0.001	−0.000326∼0.002	0.141	0.247
	CR	−0.007	−0.012∼-0.002	0.004	0.028
	CCR	0.019	0.003∼0.036	0.020	0.070
tHCY	AST	−1.673	−3.616∼0.270	0.091	0.127
	ALT	−3.387	−6.151∼-0.624	0.016	0.037
	DB	−0.069	−0.134∼-0.004	0.037	0.065
	TB	−0.228	−0.387∼-0.070	0.005	0.028
	IB	−0.155	−0.270∼-0.041	0.008	0.028
	CR	0.291	−0.130∼0.712	0.175	0.175
	CCR	−0.662	−1.585∼0.261	0.160	0.175

**TABLE 3:** GEE analysis of the associations between homocysteine and its redox forms and hepatic and renal function indicators in 39 adult patients.

Item		β	95% CI	*P* value	FDR-adjusted *P*
HCY	AST	1.139	−0.507∼2.784	0.175	0.511
	ALT	−0.356	−2.452∼1.741	0.740	0.740
	DB	−0.349	−1.213∼0.515	0.429	0.601
	TB	−0.711	−1.893∼0.471	0.238	0.511
	IB	−0.362	−1.036∼0.311	0.292	0.511
	CR	2.686	−0.668∼6.041	0.117	0.511
	CCR	−1.534	−7.067∼3.999	0.587	0.685
HHCY	AST	−56.466	−141.751∼28.820	0.194	0.272
	ALT	−139.444	−303.944∼25.055	0.097	0.272
	DB	−10.015	−31.289∼11.260	0.356	0.415
	TB	−25.504	−62.827∼11.819	0.180	0.272
	IB	−15.489	−37.929∼6.951	0.176	0.272
	CR	−0.030	−128.161∼128.100	1.000	1.000
	CCR	−178.241	−426.230∼69.748	0.159	0.272
HCY/HHCY	AST	0.013	−0.010∼0.036	0.280	0.612
	ALT	0.002	−0.017∼0.022	0.809	0.809
	DB	−0.001	−0.004∼0.002	0.421	0.612
	TB	−0.004	−0.008∼0.001	0.121	0.424
	IB	−0.002	−0.005∼0.0001	0.061	0.424
	CR	0.006	−0.019∼0.030	0.658	0.768
	CCR	0.034	−0.052∼0.120	0.437	0.612
tHCY	AST	0.030	−0.620∼0.680	0.928	0.987
	ALT	−0.009	−0.1004∼0.987	0.987	0.987
	DB	−0.063	−0.295∼0.169	0.594	0.952
	TB	0.119	−0.238∼0.476	0.514	0.952
	IB	0.182	−0.013∼0.377	0.068	0.476
	CR	0.947	−0.387∼2.281	0.164	0.574
	CCR	0.420	−1.573∼2.412	0.680	0.952

### 3.3 Inter-group difference analysis result

The comparison of tHcy-related forms between the liver and kidney injury groups and the normal function group is shown in Figure 2. At 42 h, the concentrations of Hcy (FDR-*P* = 0.039) and HHcy (FDR-*P* = 0.003) were significantly different in the kidney injury group. And HHcy was significantly elevated in the kidney injury group. At 72 h, only the Hcy level differed between the kidney injury and normal groups (FDR-*P* = 0.003), while at 96 h, a significant difference in HHcy was observed in the kidney injury group (FDR-*P*<0.001). No significant differences in tHcy-related forms were found between the liver injury and normal groups.

**FIGURE 2:**
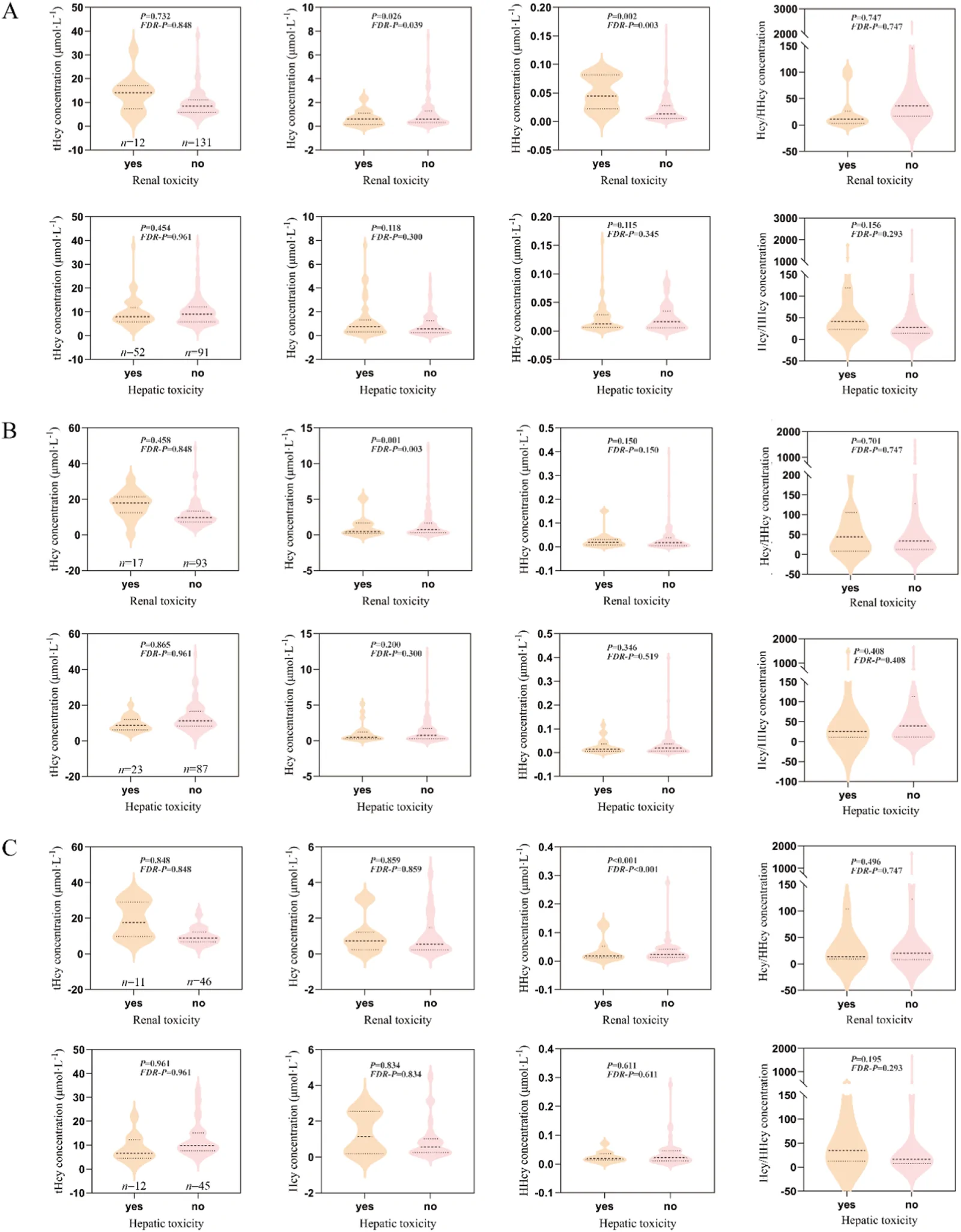
Intergroup differences in tHcy, Hcy, HHcy, and Hcy/HHcy concentrations at 42 **(A)**, 72 **(B)**, and ≥96 h **(C)** between the injury and the control group in 310 plasma samples. Center line: median; dotted lines: upper and lower quartiles.

### 3.4 ROC curve analysis results

The results of the AUC analysis are shown in Table 4. The results indicate that HHcy concentration at 42 h has good discriminative ability against nephrotoxicity, with an AUC of 0.817 (95% CI: 0.702–0.932, *P* < 0.001). tHcy concentration at 42 h also shows good discriminative ability. However, Hcy/HHcy concentration at 42 h shows the opposite discriminative ability. The original AUC was 0.232 (95% CI: 0.092–0.372, *P* = 0.002). After reversing the index direction, the adjusted AUC was 0.768 (95% CI: 0.628–0.908, *P* = 0.002) (Supplementary Table S3). At 72 h and ≥96 h, tHcy concentrations also showed good ability to differentiate nephrotoxicity, with AUCs of 0.761 (95% CI: 0.622–0.900, *P* = 0.001) and 0.812 (95% CI: 0.662–0.962, *P* = 0.001), respectively. For hepatotoxicity, the AUCs of tHcy at 72 h and ≥96 h were 0.654 (95% CI: 0.538–0.771, *P* = 0.023) and 0.715 (95% CI: 0.535–0.895, *P* = 0.023), respectively, indicating reverse differentiation ability (Supplementary Table S3). ROC analysis results are shown in Figure 3.

**TABLE 4:** ROC analysis of the discriminative ability of Hcy and its redox forms for hepatic and renal toxicity at 42 h, 72 h, and ≥96 h after HD-MTX treatment.Using “the occurrence of nephrotoxicity” as a state variable.

Time	Item	Renal toxicity					Liver toxicity				
		AUC (95%CI)	*P*	Sensitivity (%)	Specificity (%)	Threshold (μmol·L^-1^)	AUC (95%CI)	*P*	Sensitivity (%)	Specificity (%)	Threshold (μmol·L^-1^)
42 h	tHcy concentration	0.681 (0.492–0.871)	0.038	66.7	81.7	12.7	0.467 (0.368–0.566)	0.515	80.8	24.2	5.76
	Hcy concentration	0.415 (0.245–0.585)	0.329	58.3	50.4	0.60	0.560 (0.461–0.658)	0.237	67.3	47.3	0.44
	HHcy concentration	0.817 (0.702–0.932)	<0.001	66.7	87.0	0.04	0.483 (0.386–0.580)	0.737	96.2	16.5	0.003
	Hcy/HHcy concentration	0.232 (0.092–0.372)	0.002	100.0	2.3	1.38	0.578 (0.483–0.673)	0.122	80.8	44.0	20.82
72 h	tHcy concentration	0.761 (0.622–0.900)	0.001	76.5	76.3	13.49	0.346 (0.229–0.462)	0.023	100	4.6	3.57
	Hcy concentration	0.462 (0.306–0.617)	0.617	11.8	95.7	4.7	0.456 (0.334–0.579)	0.520	0.0	100	0.26
	HHcy concentration	0.508 (0.369–0.646)	0.369	88.2	25.8	0.005	0.453 (0.318–0.587)	0.485	13.0	93.1	0.078
	Hcy/HHcy concentration	0.477 (0.326–0.628)	0.326	52.9	54.8	44.05	0.482 (0.351–0.612)	0.788	91.3	19.5	9.58
≥96 h	tHcy concentration	0.812 (0.662–0.962)	0.001	63.6	93.5	15.72	0.285 (0.105–0.465)	0.023	0.0	100.0	0.66
	Hcy concentration	0.524 (0.329–0.718)	0.808	72.7	50.0	0.50	0.595 (0.400–0.791)	0.313	50.0	82.2	1.37
	HHcy concentration	0.499 (0.299–0.699)	0.992	100.0	17.4	0.007	0.441 (0.276–0.606)	0.531	91.7	22.2	0.008
	Hcy/HHcy concentration	0.488 (0.297–0.679)	0.903	45.5	67.4	38.41	0.619 (0.454–783)	0.210	91.7	35.6	11.34

**FIGURE 3:**
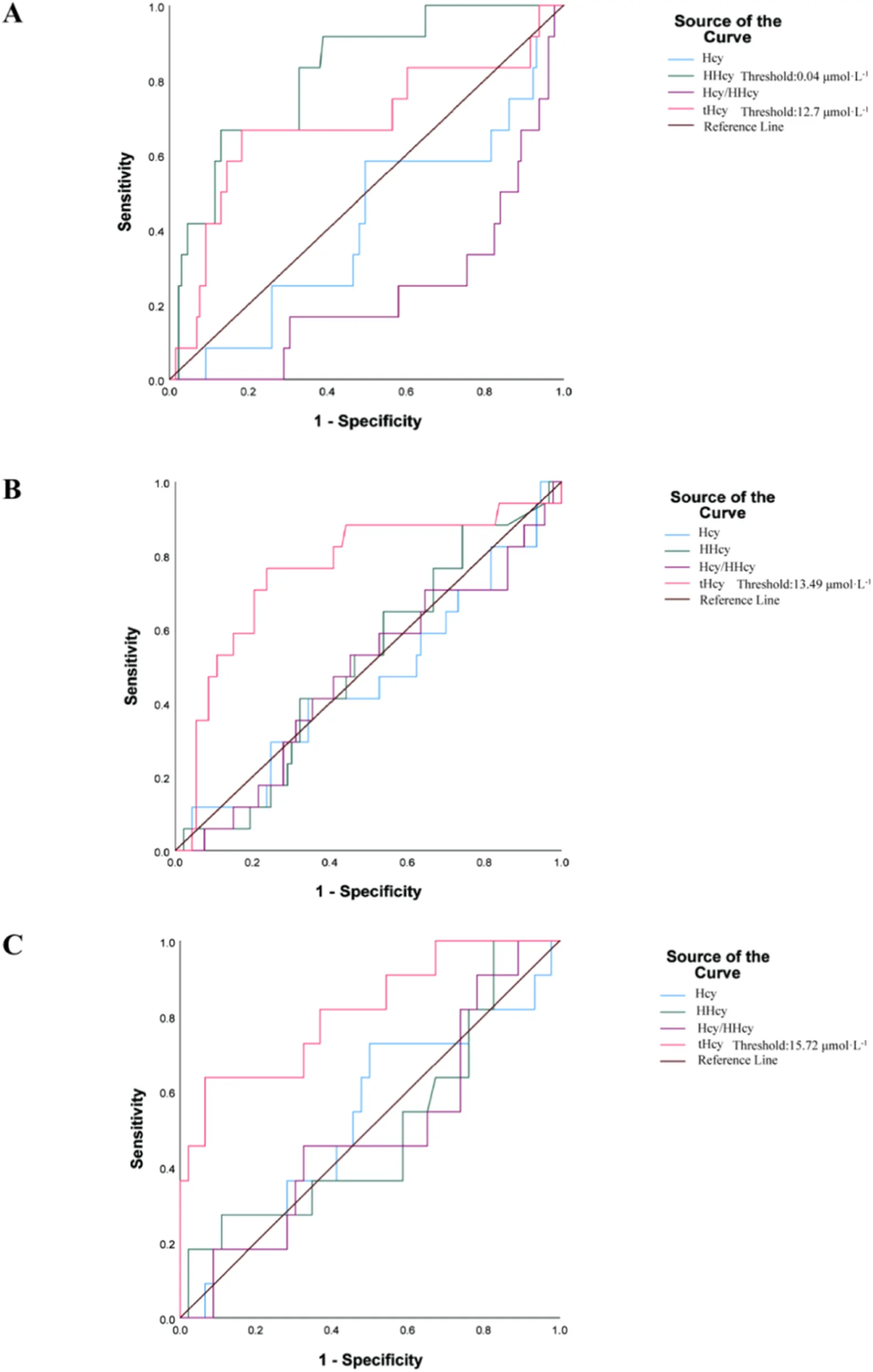
ROC curves for the discriminative ability of Hcy and its redox forms for nephrotoxicity at 42 h **(A)**, 72 h **(B)**, and ≥96 h **(C)** after HD-MTX treatment, based on 310 plasma samples.

## 4 Discussion

### 4.1 The correlation between toxic reactions and concentration

Intracellular biological thiols play a crucial role in counteracting oxidative stress and maintaining redox homeostasis by regulating the redox state between reductive thiols and their oxidized disulfide forms (Dong et al., 2023). Studies suggest tHcy may be implicated in liver toxicity in children with ALL and exhibits transient elevation following HD-MTX chemotherapy (Kubota et al., 2014; Kishi et al., 2023). Similarly, the GEE analysis results of the pediatric subgroup in our study showed that tHcy concentration was significantly negatively associated with ALT (95%CI:-6.151∼-0.624), TB (-0.387∼-0.070), and DB (-0.270∼-0.041) (FDR-P<0.05), while no similar association was observed in adult patients. It has been reported that elevated tHcy levels are significantly associated with renal impairment (Chen et al., 2016), but no significant association was found between tHcy and CR and CCR in our study. This difference may be due to the limited sample size. Furthermore, this analysis did not include baseline total homocysteine levels; further validation through an expanded sample size is needed in the future.

Currently, there are no reports on the association between various redox forms of tHcy and hepatotoxicity and nephrotoxicity. Our study is the first to find a significant negative correlation between Hcy/HHcy and the renal function indicator CR in pediatric patients. (*P* = 0.004, FDR-*P* = 0.028, 95%CI:-0.012∼-0.002) (see Table 2). This result suggests that HHcy may be associated with renal function changes in pediatric patients following HD-MTX treatment, although this finding requires further validation in independent cohorts. Monitoring tHcy-related redox forms may provide clinicians with complementary information and may help discriminate HD-MTX-related renal toxicity.

### 4.2 Cross-sectional distribution of tHcy, Hcy, HHcy, and Hcy/HHcy concentrations at different sampling time points

Studies have shown that tHcy levels initially increase within 24 h, followed by a slight decrease between 48 and 72 h (Kubota et al., 2014). Consistently, our study observed that tHcy levels increased from 42 to 72 h and then decreased between 72 and 96 h (Figure 4). It has been reported that tHcy may contribute to tissue injury by increasing the generation of ROS and promoting oxidative stress and inflammatory responses (Yuan et al., 2023). Studies have found that patients with chronic kidney damage exhibit higher oxidative stress levels and lower antioxidant enzyme activity (Chen et al., 2016). Although these findings were mainly derived from populations with chronic kidney disease and cannot be directly extrapolated to acute kidney injury associated with HD-MTX treatment, they suggest that impaired renal function may be associated with alterations in systemic redox homeostasis. In this context, we further characterized the distribution of Hcy and HHcy across different sampling time points after HD-MTX administration (Figure 4). It should be noted that the results shown in Figure 4 represent cross-sectional summaries at different sampling time points rather than continuous longitudinal changes within the same patients. The paired-sample analysis at 42 h, 72 h, and 96 h after HD-MTX treatment is presented in Supplementary Figure S2.

**FIGURE 4:**
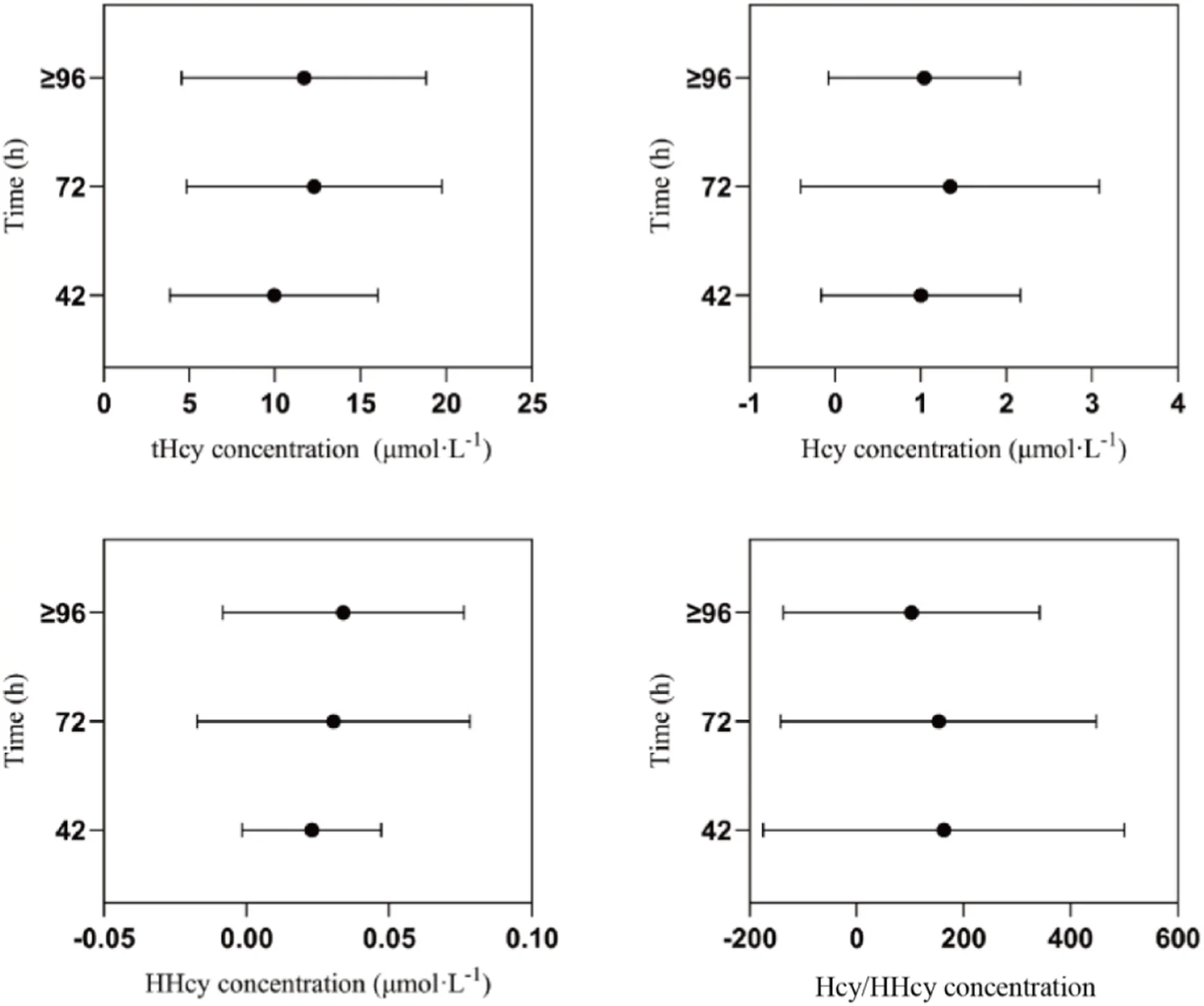
Cross-sectional distribution of tHcy, Hcy, HHcy, and Hcy/HHcy concentrations at different sampling time points after HD-MTX administration. The solid dots: mean values, error bars: standard deviation (mean ± SD).

The concentration changes observed between 42 and 72 h may be attributed to the inhibitory effect of HD-MTX on dihydrofolate reductase, which interferes with folate metabolism and the remethylation of tHcy to methionine, thereby promoting the elevation of tHcy and its redox forms (Hcy and HHcy) in the early phase of treatment. A portion of Hcy is converted to HHcy under oxidative conditions, leading to a rapid increase in HHcy. Following the initiation of calcium folinate (CF) rescue therapy after 72–96 h, methionine cycle function gradually recovers and tHcy production decreases, which may explain the sustained decline in tHcy levels after 72–96 h. However, in our study, most patients reach therapeutic targets within 42 h, after which leucovorin rescue could be discontinued, while other patients requiring prolonged rescue generally have higher MTX concentrations, even with delayed MTX clearance. This may result in prolonged exposure to relatively high concentrations of MTX, thereby increasing drug toxicity and further exacerbating oxidative stress. Persistent oxidative stress may continuously oxidize the remaining reduced Hcy to HHcy, thereby maintaining a slow upward trend in HHcy levels. Meanwhile, as tHcy production decreases with the progressive recovery of methionine cycle function, the substrate supply for maintaining reduced Hcy levels is reduced. The remaining Hcy is continuously oxidized to HHcy, contributing to the decline in Hcy levels during the later phase (72–96 h) and ultimately leading to a sustained decrease in the Hcy/HHcy. This trend is consistent with previous reports (Dong et al., 2023).

However, since oxidative stress levels were not directly measured in this study, the above mechanisms remain hypothetical and require further investigation with an expanded sample size in future studies.

### 4.3 Analysis of differences between groups

Elevated tHcy levels can induce excessive free radical production through their own oxidation, thereby triggering oxidative stress (Huang et al., 2019). Studies have shown that tHcy concentration levels were significantly decreased in the liver injury group and significantly increased in the kidney injury group (*P* < 0.05) (Mahmoud et al., 2018; Huang et al., 2019). Consistent with the overall trend of previous studies, our study found that tHcy levels decreased after HD-MTX treatment in patients with liver injury, while tHcy concentrations increased at 42, 72, and 96 h in patients with kidney injury. However, after adjusting for intra-patient repeated measures and covariates such as height, weight, and sex using a GEE model, no statistically significant differences were found between the liver and kidney injury groups and the normal group at any observation time point. This suggests that changes in tHcy may be associated with HD-MTX-related liver and kidney injury, but the differences between groups may be affected by individual differences and other clinical factors, requiring further validation in larger-scale studies.

Studies have found that MTX can reduce total antioxidant capacity and increase the disulfide bond/total thiol ratio (Yanaşoğlu et al., 2022), and its induced hepatotoxicity and nephrotoxicity are closely associated with oxidative damage and inflammatory processes (Deniz et al., 2025; Hassanein et al., 2023; Hassanein et al., 2019; Morsy et al., 2022). Previous studies on HD-MTX have mainly focused tHcy levels, with relatively limited attention paid to different redox forms of Hcy and their relative changes. Our study further measured both reduced Hcy and oxidized HHcy simultaneously, and used the Hcy/HHcy to estimate the redox status of Hcy, while also observing its relationship with HD-MTX-related nephrotoxicity at different time points. GEE analysis showed that Hcy (FDR-*P* = 0.039) and HHcy (FDR-*P* = 0.003) levels at 42 h were significantly different between the renal injury group and the normal group, and the Hcy/HHcy at 42 h was significantly negatively correlated with CR. Furthermore, significant inter-group differences in Hcy at 72 h and Hcy/HHcy at 96 h and beyond were still observed in the renal injury group. In contrast, no significant differences in tHcy-related indicators were observed between the liver injury group and the normal liver function group. These results suggest that HD-MTX-related nephrotoxicity may be related to time-dependent changes in tHcy-related redox forms. In addition to changes in tHcy levels, changes in Hcy and HHcy may also provide Supplementary Material for evaluating HD-MTX-related renal function impairment. In particular, changes in Hcy and HHcy levels in the early post-treatment period warrant further investigation and validation in future studies.

### 4.4 ROC curve analysis

The value of tHcy as a disease-related biomarker may vary depending on the target organ, disease stage, and timing of monitoring. The literature (Li et al., 2022) indicates that plasma tHcy levels are independently and positively correlated with the risk of advanced liver fibrosis in non-alcoholic fatty liver disease (NAFLD), demonstrating its potential value as a biomarker for predicting NAFLD liver fibrosis (AUC = 0.7319, *P* < 0.05). Furthermore, in our study, tHcy demonstrated a reverse discriminatory ability for hepatotoxicity at 72 h and ≥96 h (72 h: AUC = 0.654, 95% CI: 0.538–0.771, *P* = 0.023; ≥96 h: AUC = 0.715, 95% CI: 0.535–0.895, *P* = 0.023) (Supplementary Table S3), meaning that lower tHcy levels were actually associated with the occurrence of hepatotoxicity. This is consistent with previous reports of lower tHcy levels in cases of liver injury (Mahmoud et al., 2018). However, our study is currently an exploratory analysis, and further biomarker research will be needed in the future by expanding the sample size.

Regarding renal impairment, tHcy metabolism is closely linked to renal function status. tHcy is almost entirely reabsorbed from primary urine by renal tubules, with its clearance rate decreasing by about 30% in chronic kidney disease (CKD) (Kruglova et al., 2023). It has been reported that Hcy concentration shows good diagnostic predictive value in CKD stage II (AUC = 0.848), but its predictive efficacy is not significant in stages IIIa-IIIb (AUC = 0.567) (Kruglova et al., 2023). In our study, at 42 h, HHcy (AUC = 0.817, 95% CI: 0.702–0.932, P < 0.001), tHcy (AUC = 0.681, 95% CI: 0.492–0.871, *P* = 0.038) (Table 4), and the Hcy/HHcy (AUC = 0.768, 95% CI: 0.628–0.908, *P* = 0.002) (Supplementary Table S3) all demonstrated good discriminatory ability, suggesting that during the early phase of HD-MTX chemotherapy (42 h), tHcy-related forms may be associated with the development of nephrotoxicity. However, as time progressed to 72 h and ≥96 h, the discriminatory ability of HHcy and the Hcy/HHcy declined significantly (AUC <0.5), while only tHcy maintained relatively stable discriminatory ability (72 h: AUC = 0.761, 95% CI: 0.622–0.900, *P* = 0.001; ≥96 h: AUC = 0.812, 95% CI: 0.662–0.962, *P* = 0.001). This may suggest that different forms of homocysteine exhibit distinct discriminatory patterns at different stages of the development and progression of HD-MTX-associated kidney injury. Specifically, the discriminatory patterns of HHcy and Hcy/HHcy at earlier time points may be related to changes in the redox state of homocysteine and potential oxidative stress processes during the early stages of HD-MTX treatment, whereas the continued discriminatory ability of tHcy at later time points may more closely reflect changes in overall Hcy levels reflecting alterations in Hcy metabolism and clearance associated with renal dysfunction. The exploratory analysis in this study aims to draw clinical attention to the different forms of tHcy in HD-MTX-induced nephrotoxicity and to provide preliminary evidence for subsequent mechanistic studies and prospective validation.

### 4.5 Limitations

This study has several limitations.

First, because the tHcy-related analyses were performed using residual blood samples collected during routine MTX therapeutic drug monitoring, baseline tHcy, Hcy, and HHcy levels before HD-MTX administration were not available. Therefore, the post-treatment differences in tHcy-related forms may partly reflect HD-MTX-related changes and may also be influenced by interindividual variation. The present findings should therefore be considered exploratory and require further validation.

Second, the relatively small sample size and heterogeneous clinical background (including comorbidities, concomitant medications, and drug interactions) may have influenced the observed associations. Although we adjusted for height, weight, age, sex, baseline renal and hepatic function, MTX concentration, HD-MTX dose, and delayed MTX elimination, some potential confounders were not systematically collected, including baseline homocysteine levels and related metabolic indicators. In this study, no patients received concomitant medications with hepatotoxic or nephrotoxic effects, and none took folic acid or vitamin B12 during the treatment period. Future studies with an expanded sample size are needed for further investigation.

Third, the cross-sectional nature of the multi-time-point data—with decreasing sample sizes over time and incomplete patient overlap across time points—limited the interpretation of the observed trends as true longitudinal changes within individuals, and may have introduced selection bias at later time points.

Fourth, this study was exploratory in nature. The limited number of nephrotoxicity events (12 at 42 h, 17 at 72 h, and 11 at ≥96 h) and the lack of internal validation (e.g., bootstrap) for the ROC analyses may have affected the stability of the AUC estimates. In addition, the use of CTCAE ≥1 as the toxicity threshold may have included mild or transient laboratory abnormalities; however, because the number of nephrotoxicity events was too small, it was not appropriate to further adjust the grading criteria or stratify by severity of organ dysfunction. Consequently, a more detailed assessment of nephrotoxicity severity could not be performed.

In summary, future prospective studies with larger sample sizes, standardized baseline and serial post-treatment measurements, more comprehensive confounder adjustment, and more detailed toxicity grading are warranted to validate our findings.

## 5 Conclusion

In conclusion, this study demonstrates that redox forms of homocysteine are differentially associated with hepatotoxicity and nephrotoxicity following HD-MTX chemotherapy. In pediatric patients, the Hcy/HHcy was correlated with CR, while tHcy was correlated with ALT, DB, and TB. Furthermore, significant between-group differences in Hcy and HHcy were observed in nephrotoxicity at multiple time points. ROC analyses further supported the discriminatory ability of tHcy, HHcy, and the Hcy/HHcy at 42 h for renal injury, whereas tHcy exhibited reverse discriminatory ability for hepatotoxicity at later time points. These findings suggest that, beyond tHcy levels, monitoring the redox forms of tHcy may have potential clinical value, and that HHcy and the Hcy/HHcy may be associated with HD-MTX-associated nephrotoxicity and may have potential discriminatory value for renal injury.

## Data Availability

The raw data supporting the conclusions of this article will be made available by the authors, without undue reservation.
