# Supplementary material for: The relationship of homocysteine and its redox forms with renal toxicity after high-dose methotrexate treatment in patients with leukemia or lymphoma
Source: Front Pharmacol. 2026 Oct 20;17:1972047. doi: 10.3389/fphar.2026.1972047 (PMC13727596; doi:10.3389/fphar.2026.1972047)
Supplement: Supplementary file 2 [file Image2.pdf]

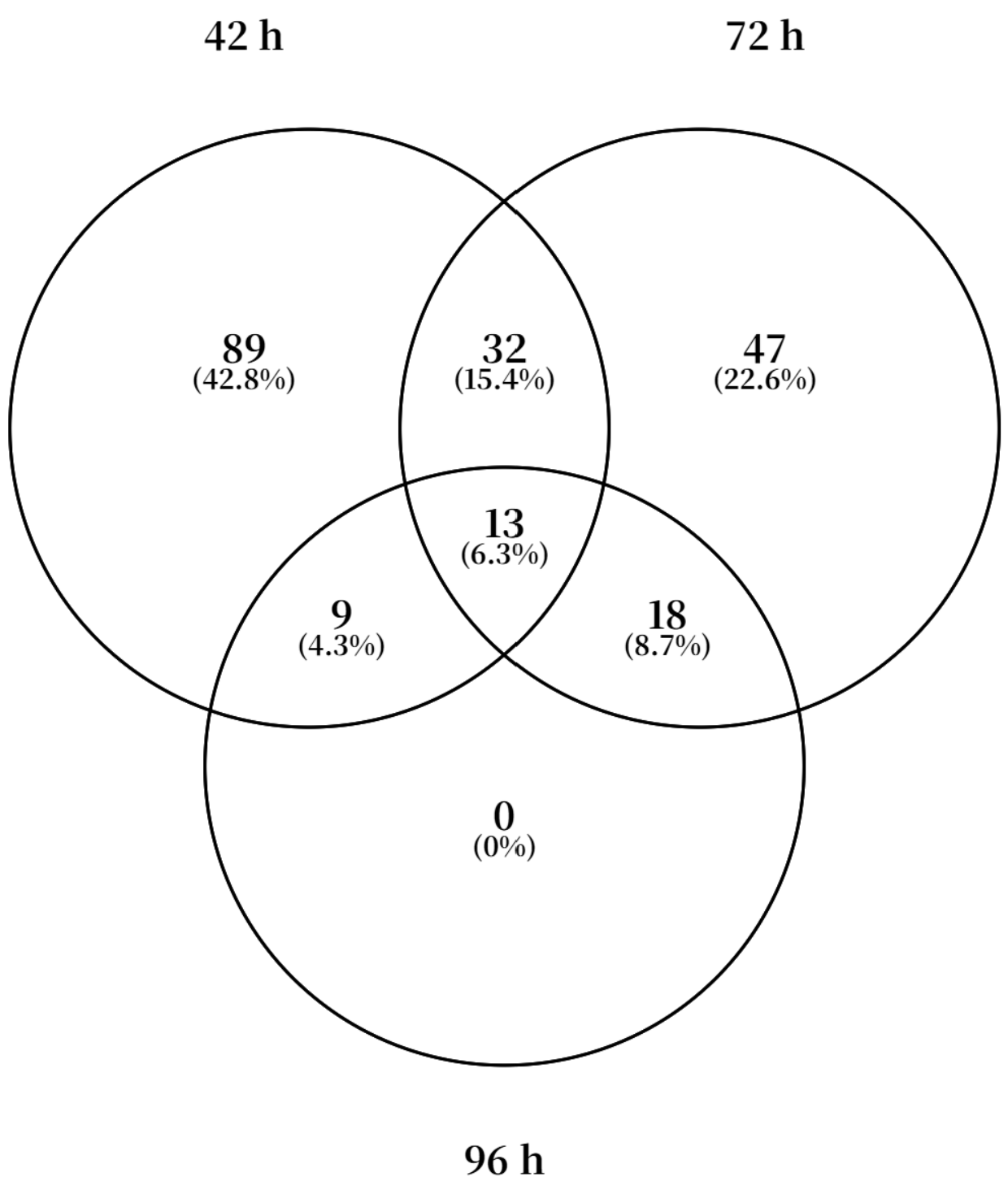

Supplementary Figure 2 Paired-sample analysis results at 42, 72, and 96 h after HD-MTX treatment.
