# Supplementary material for: The relationship of homocysteine and its redox forms with renal toxicity after high-dose methotrexate treatment in patients with leukemia or lymphoma
Source: Front Pharmacol. 2026 Oct 20;17:1972047. doi: 10.3389/fphar.2026.1972047 (PMC13727596; doi:10.3389/fphar.2026.1972047)
Supplement: Supplementary file 3 [file Table1.docx]

| Characteristic | Nephrotoxicity (*n* = 40)  Mean±SD | No nephrotoxicity (*n* =270)  Mean±SD |
| --- | --- | --- |
| Age (years) | 36.35±19.19 | 21.02±19.01 |
| Sex (male/female) | 30/10 | 168/102 |
| MTX concentration (μmol·L^-1^) | 0.47±0.91 | 0.56±2.37 |
| leucovorin rescue dose (mg) | 47.92 ± 52.38 | 39.15 ± 57.54 |
| Baseline creatinine (CR) | 66.45±39.61 | 43.11±14.98 |
| Baseline CCR | 135.40±50.27 | 139.94±36.77 |
| Baseline AST | 25.36±14.81 | 36.39±36.52 |
| Baseline ALT | 30.94±14.10 | 37.34±27.24 |
| Baseline TB | 15.06±11.42 | 12.39±7.88 |
| Baseline DB | 6.14±7.37 | 5.47±4.31 |
| Baseline IB | 9.13±4.68 | 8.26±4.07 |

****Supplementary Table 1** Baseline and clinical characteristics of patients with and without renal toxicity after HD-MTX treatment**
