# Supplementary material for: The relationship of homocysteine and its redox forms with renal toxicity after high-dose methotrexate treatment in patients with leukemia or lymphoma
Source: Front Pharmacol. 2026 Oct 20;17:1972047. doi: 10.3389/fphar.2026.1972047 (PMC13727596; doi:10.3389/fphar.2026.1972047)
Supplement: Supplementary file 4 [file Table2.docx]

| Characteristic | Hepatotoxicity (*n* = 87)  Mean±SD | No hepatotoxicity (n =223)  Mean±SD |
| --- | --- | --- |
| Age (years) | 16.51±15.64 | 28.29±21.06 |
| Sex (male/female) | 45/42 | 153/70 |
| MTX concentration (μmol·L^-1^) | 0.78±2.95 | 0.36±1.07 |
| leucovorin rescue dose (mg) | 45.87 ± 57.37 | 36.74 ± 54.02 |
| Baseline creatinine (CR) | 42.97±28.56 | 48.63±11.72 |
| Baseline CCR | 146.38±40.08 | 133.58±36.59 |
| Baseline AST | 45.22±35.33 | 26.66±4.24 |
| Baseline ALT | 45.48±36.06 | 27.74±6.81 |
| Baseline TB | 16.75±10.8 | 9.41±3.07 |
| Baseline DB | 7.77±6.37 | 3.72±1.08 |
| Baseline IB | 10.11±5.37 | 6.93±1.83 |

****Supplementary Table 2** Baseline and clinical characteristics of patients with and without hepatic toxicity after HD-MTX treatment**
