# Supplementary material for: The relationship of homocysteine and its redox forms with renal toxicity after high-dose methotrexate treatment in patients with leukemia or lymphoma
Source: Front Pharmacol. 2026 Oct 20;17:1972047. doi: 10.3389/fphar.2026.1972047 (PMC13727596; doi:10.3389/fphar.2026.1972047)
Supplement: Supplementary file 5 [file Table3.docx]

| Time | Item | Renal toxicity | | | | | Liver toxicity | | | | |
| --- | --- | --- | --- | --- | --- | --- | --- | --- | --- | --- | --- |
|  |  | AUC (95%CI) | *P* | Sensitivity(%) | Specificity(%) | Threshold(μmol·L^-1^) | AUC (95%CI) | *P* | Sensitivity(%) | Specificity(%) | Threshold(μmol·L^-1^) |
| 42h | tHcy concentration | 0.319  (0.129-0.508) | 0.038 | 91.6 | 16.7 | 4.57 | 0.533  (0.434-0.632) | 0.515 | 46.3 | 65.8 | 9.8 |
|  | Hcy  concentration | 0.585  (0.415-0.755) | 0.329 | 81.7 | 41.7 | 0.23 | 0.440  (0.342-0.539) | 0.237 | 55.2 | 48.7 | 0.571 |
|  | HHcy concentration | 0.183  (0.068-0.298) | <0.001 | 2.3 | 100 | 0.088 | 0.517  (0.420-0.614) | 0.737 | 7.5 | 96.1 | 0.036 |
|  | Hcy/ HHcy concentration | 0.768  (0.628-0.908) | 0.002 | 75.6 | 75.0 | 16.69 | 0.422  (0.327-0.517) | 0.122 | 14.9 | 93.4 | 66.80 |
| 72h | tHcy concentration | 0.239  (0.100-0.378) | 0.001 | 76.5 | 23.5 | 11.8 | 0.654  (0.538-0.771) | 0.023 | 76.70 | 56.2 | 8.57 |
|  | Hcy  concentration | 0.538  (0.383-0.694) | 0.617 | 62.4 | 52.9 | 0.474 | 0.544  (0.421-0.666) | 0.520 | 80.2 | 23.2 | 0.35 |
|  | HHcy concentration | 0.492  (0.354-0.631) | 0.921 | 19.4 | 94.1 | 0.0471 | 0.547  (0.413-0.682) | 0.485 | 56.4 | 67.9 | 0.012 |
|  | Hcy/ HHcy concentration | 0.523  (0.372-0.674) | 0.763 | 86 | 29.4 | 8.81 | 0.518  (0.388-0.649) | 0.788 | 14.2 | 82.5 | 12.3 |
| ≥96h | tHcy concentration | 0.188  (0.038-0.338-) | 0.001 | 0 | 100 | 34.81 | 0.715  (0.535-0.895) | 0.023 | 87.3 | 13.6 | 3.67 |
|  | Hcy  concentration | 0.476  (0.282-0.671) | 0.808 | 93.5 | 18.2 | 0.116 | 0.405  (0.209-0.6) | 0.313 | 63.2 | 24.7 | 0.86 |
|  | HHcy concentration | 0.501  (0.301-0.701) | 0.992 | 58.7 | 63.6 | 0.0199 | 0.559  (0.394-0.724) | 0.531 | 25.6 | 85.4 | 0.007 |
|  | Hcy/ HHcy concentration | 0.512  (0.321-0.703) | 0.903 | 65.2 | 54.5 | 14.03 | 0.381  (0.217-0.546) | 0.210 | 35.6 | 80.4 | 23.4 |

**Supplementary Table 3** ROC analysis of the discriminative ability of Hcy and its redox forms for hepatic and renal toxicity at 42 h, 72 h, and ≥96 h after HD-MTX treatment. Using “no nephrotoxicity” as a state variable
